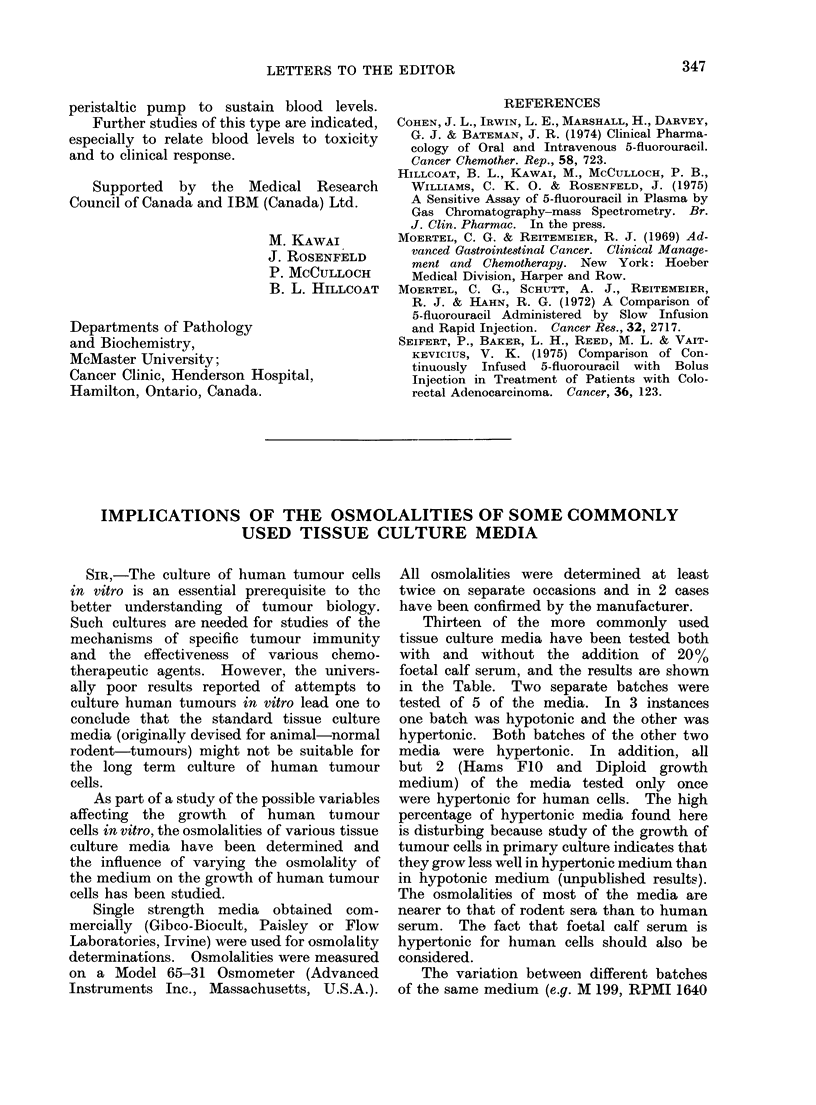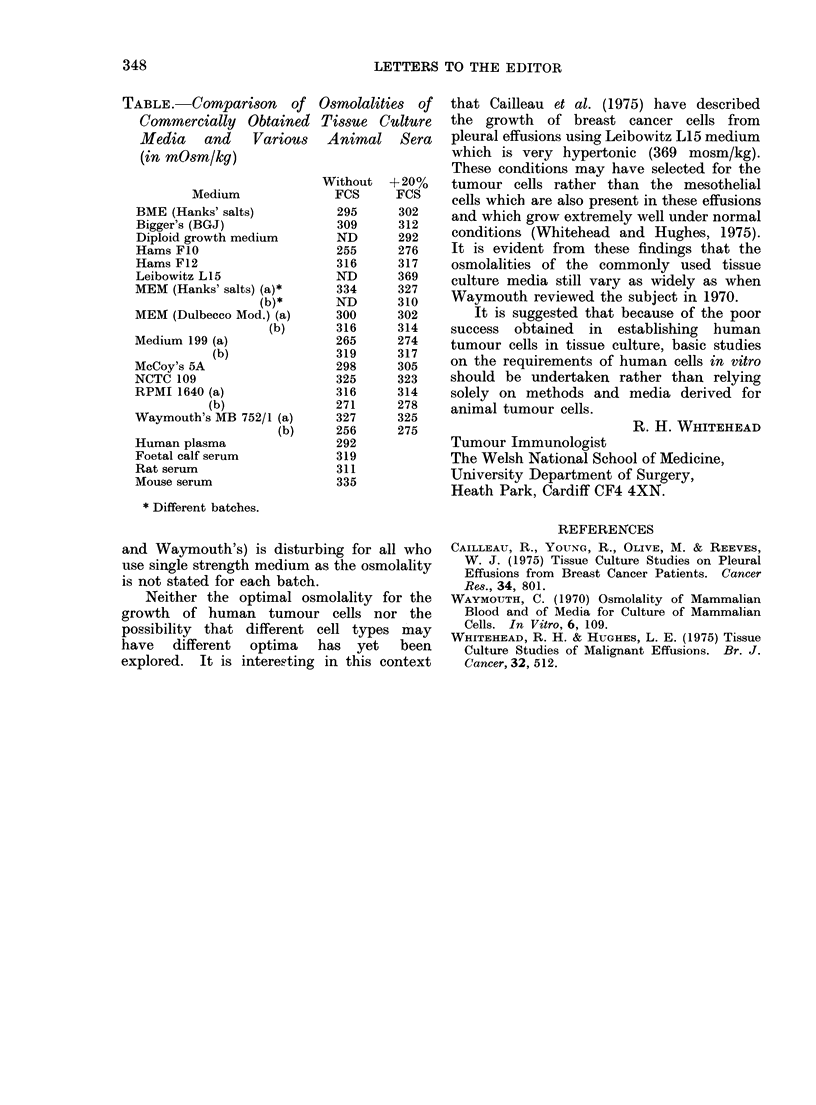# Letter: Implications of the osmolalities of some commonly used tissue culture media.

**DOI:** 10.1038/bjc.1976.52

**Published:** 1976-03

**Authors:** R. H. Whitehead


### IMPLICATIONS OF THE OSMOLALITIES OF SOME COMMONLY USED TISSUE CULTURE MEDIA

SIR,—The culture of human tumour cells *in vitro* is an essential prerequisite to the better understanding of tumour biology. Such cultures are needed for studies of the mechanisms of specific tumour immunity and the effectiveness of various chemotherapeutic agents. However, the universally poor results reported of attempts to culture human tumours *in vitro* lead one to conclude that the standard tissue culture media (originally devised for animal—normal rodent—tumours) might not be suitable for the long term culture of human tumour cells.

As part of a study of the possible variables affecting the growth of human tumour cells *in vitro*, the osmolalities of various tissue culture media have been determined and the influence of varying the osmolality of the medium on the growth of human tumour cells has been studied.

Single strength media obtained commercially (Gibco-Biocult, Paisley or Flow Laboratories, Irvine) were used for osmolality determinations. Osmolalities were measured on a Model 65–31 Osmometer (Advanced Instruments Inc., Massachusetts, U.S.A.). All osmolalities were determined at least twice on separate occasions and in 2 cases have been confirmed by the manufacturer.

Thirteen of the more commonly used tissue culture media have been tested both with and without the addition of 20% foetal calf serum, and the results are shown in the Table. Two separate batches were tested of 5 of the media. In 3 instances one batch was hypotonic and the other was hypertonic. Both batches of the other two media were hypertonic. In addition, all but 2 (Hams F10 and Diploid growth medium) of the media tested only once were hypertonic for human cells. The high percentage of hypertonic media found here is disturbing because study of the growth of tumour cells in primary culture indicates that they grow less well in hypertonic medium than in hypotonic medium (unpublished results). The osmolalities of most of the media are nearer to that of rodent sera than to human serum. The fact that foetal calf serum is hypertonic for human cells should also be considered.

The variation between different batches of the same medium (*e.g.* M 199, RPMI 1640

TABLE.—*Comparison of Osmolalities of Commercially Obtained Tissue Culture Media and Various Animal Sera (in mOsm/kg)*

| Medium | Without FCS | +20% FCS |
|---|---|---|
| BME (Hanks' salts) | 295 | 302 |
| Bigger's (BGJ) | 309 | 312 |
| Diploid growth medium | ND | 292 |
| Hams F10 | 255 | 276 |
| Hams F12 | 316 | 317 |
| Leibowitz L15 | ND | 369 |
| MEM (Hanks' salts) (a)* | 334 | 327 |
| (b)* | ND | 310 |
| MEM (Dulbecco Mod.) (a) | 300 | 302 |
| (b) | 316 | 314 |
| Medium 199 (a) | 265 | 274 |
| (b) | 319 | 317 |
| McCoy's 5A | 298 | 305 |
| NCTC 109 | 325 | 323 |
| RPMI 1640 (a) | 316 | 314 |
| (b) | 271 | 278 |
| Waymouth's MB 752/1 (a) | 327 | 325 |
| (b) | 256 | 275 |
| Human plasma | 292 | |
| Foetal calf serum | 319 | |
| Rat serum | 311 | |
| Mouse serum | 335 | |

* Different batches.

and Waymouth's) is disturbing for all who use single strength medium as the osmolality is not stated for each batch.

Neither the optimal osmolality for the growth of human tumour cells nor the possibility that different cell types may have different optima has yet been explored. It is interesting in this context that Cailleau *et al.* (1975) have described the growth of breast cancer cells from pleural effusions using Leibowitz L15 medium which is very hypertonic (369 mosm/kg). These conditions may have selected for the tumour cells rather than the mesothelial cells which are also present in these effusions and which grow extremely well under normal conditions (Whitehead and Hughes, 1975). It is evident from these findings that the osmolalities of the commonly used tissue culture media still vary as widely as when Waymouth reviewed the subject in 1970.

It is suggested that because of the poor success obtained in establishing human tumour cells in tissue culture, basic studies on the requirements of human cells *in vitro* should be undertaken rather than relying solely on methods and media derived for animal tumour cells.

R. H. WHITEHEAD

Tumour Immunologist
The Welsh National School of Medicine,
University Department of Surgery,
Heath Park, Cardiff CF4 4XN.

REFERENCES

CAILLEAU, R., YOUNG, R., OLIVE, M. & REEVES, W. J. (1975) Tissue Culture Studies on Pleural Effusions from Breast Cancer Patients. *Cancer Res.*, **34**, 801.

WAYMOUTH, C. (1970) Osmolality of Mammalian Blood and of Media for Culture of Mammalian Cells. *In Vitro*, **6**, 109.

WHITEHEAD, R. H. & HUGHES, L. E. (1975) Tissue Culture Studies of Malignant Effusions. *Br. J. Cancer*, **32**, 512.